# *PIGN* gene expression aberration is associated with genomic instability and leukemic progression in acute myeloid leukemia with myelodysplastic features

**DOI:** 10.18632/oncotarget.15136

**Published:** 2017-02-07

**Authors:** Emmanuel K. Teye, Abigail Sido, Ping Xin, Niklas K. Finnberg, Prashanth Gokare, Yuka I. Kawasawa, Anna C. Salzberg, Sara Shimko, Michael Bayerl, W. Christopher Ehmann, David F. Claxton, Witold B. Rybka, Joseph J. Drabick, Hong-Gang Wang, Thomas Abraham, Wafik S. El-Deiry, Robert A. Brodsky, Raymond J. Hohl, Jeffrey J. Pu

**Affiliations:** ^1^ Penn State Hershey Cancer Institute, Penn State University College of Medicine, Hershey, Pennsylvania, USA; ^2^ Department of Medicine, Penn State University College of Medicine, Hershey, Pennsylvania, USA; ^3^ Department of Hematology/Oncology, Fox Chase Cancer Center, Philadelphia, Pennsylvania, USA; ^4^ Institute for Personalized Medicine, Penn State University College of Medicine, Hershey, Pennsylvania, USA; ^5^ Department of Pharmacology, Penn State University College of Medicine, Hershey, Pennsylvania, USA; ^6^ Department of Biochemistry and Molecular Biology, Penn State University College of Medicine, Hershey, Pennsylvania, USA; ^7^ Department of Pathology, Penn State University College of Medicine, Hershey, Pennsylvania, USA; ^8^ Department of Pediatrics, Penn State University College of Medicine, Hershey, Pennsylvania, USA; ^9^ Department of Neural and Behavioral Science, Pennsylvania State University, Hershey, Pennsylvania, USA; ^10^ Microscopy Imaging Facility, Pennsylvania State University, Hershey, Pennsylvania, USA; ^11^ Division of Hematology, Department of Medicine, Johns Hopkins University School of Medicine, Baltimore, Maryland, USA

**Keywords:** PIGN gene expression aberration, MDS, AML with myelodysplasia-related changes (AML-MRC), genomic instability, leukemogenesis

## Abstract

Previous studies have linked increased frequency of glycosylphosphatidylinositol-anchor protein (GPI-AP) deficiency with genomic instability and the risk of carcinogenesis. However, the underlying mechanism is still not clear. A randomForest analysis of the gene expression array data from 55 MDS patients (GSE4619) demonstrated a significant (p = 0.0007) correlation (Pearson r =-0.4068) between GPI-anchor biosynthesis gene expression and genomic instability, in which *PIGN*, a gene participating in GPI-AP biosynthesis, was ranked as the third most important in predicting risk of MDS progression. Furthermore, we observed that *PIGN* gene expression aberrations (increased transcriptional activity but diminished to no protein production) were associated with increased frequency of GPI-AP deficiency in leukemic cells during leukemic transformation/progression. *PIGN* gene expression aberrations were attributed to partial intron retentions between exons 14 and 15 resulting in frameshifts and premature termination which were confirmed by examining the RNA-seq data from a group of AML patients (phs001027.v1.p1). *PIGN* gene expression aberration correlated with the elevation of genomic instability marker expression that was independent of the *TP53* regulatory pathway. Suppression/elimination of PIGN protein expression caused a similar pattern of genomic instability that was rescued by PIGN restoration. Finally, we found that PIGN bound to the spindle assembly checkpoint protein, MAD1, and regulated its expression during the cell cycle. In conclusion, *PIGN* gene is crucial in regulating mitotic integrity to maintain chromosomal stability and prevents leukemic transformation/progression.

## INTRODUCTION

Myelodysplastic syndromes (MDS) are a heterogeneous collection of clonal hematological malignancies that affect about 13,000 people annually in the United States alone with about a one-third propensity of progression into acute myeloid leukemia (AML) [1]. MDS is conventionally classified as AML with myelodysplasia-related changes (AML-MRC) when blood or bone marrow blast populations reach or exceed 20% with dysplastic morphology in 50% or more cells in more than two myeloid lineages [2, 3]. AML is more aggressive and molecularly diverse, involving an unconstrained proliferation of aberrant myeloid progenitor cells. These aberrant myeloid progenitor cells possess genetic aberrations, populate the bone marrow and peripheral blood, and contribute to leukemia progression by driving clonal evolution [4].

Genomic instability is associated with cancer initiation and progression and has been indicated as a driver of the clonal evolution of MDS to AML [5–8]. Genomic instability is responsible for the accumulation of genetic abnormalities that contribute to the transformation of MDS into AML [5, 9]. In fact, the frequency of cytogenetic aberrations at the initial presentation of MDS is less than 50% but this frequency increases with progression due to loss or gain of large chromosomal segments [10, 11]. Previous studies have associated genomic instability with increased frequency of glycosylphosphatidylinositol-anchor protein (GPI-AP) deficiency [12–15]. Moreover, multiple studies have proposed GPI-AP loss as a predictor of leukemic transformation and have linked increased frequency of GPI-AP deficiency to genomic instability [15–17]. However, the biomarker and the underlying mechanism that link GPI-AP loss to genomic instability and leukemic transformation are yet to be elucidated. Recently, a gene called Phosphatidylinositol Glycan Anchor Biosynthesis; Class N (*PIGN*), which is located at the 18q21.33 locus, was suggested as a cancer chromosomal instability (CIN) suppressor in a colon cancer model [18]. The *PIGN* gene encodes a phosphoethanolamine (EtNP) transferase involved in the terminal steps of GPI-AP anchor biosynthesis [19, 20]. Germline mutations in the *PIGN* gene have been implicated in GPI-AP deficiency and are associated with multiple congenital anomalies and developmental defects [19, 21–34]. Interestingly, CIN, a form of genomic instability, has been linked with risk of leukemic transformation of MDS and is associated with poor overall survival in MDS patients [35]. However, no literature has yet addressed the role of the *PIGN* gene in hematological malignancy formation and progression. This study investigated the relationship between *PIGN* gene expression aberration, genomic instability, and leukemic transformation/progression. We showed for the first time that PIGN plays a vital role in maintaining chromosomal stability and preventing leukemic transformation/progression in a subgroup of patients with MDS or AML-MRC.

## RESULTS

### *PIGN* gene expression profile links to genomic stability, especially MDS progression risk stratification

**Figure 1:**
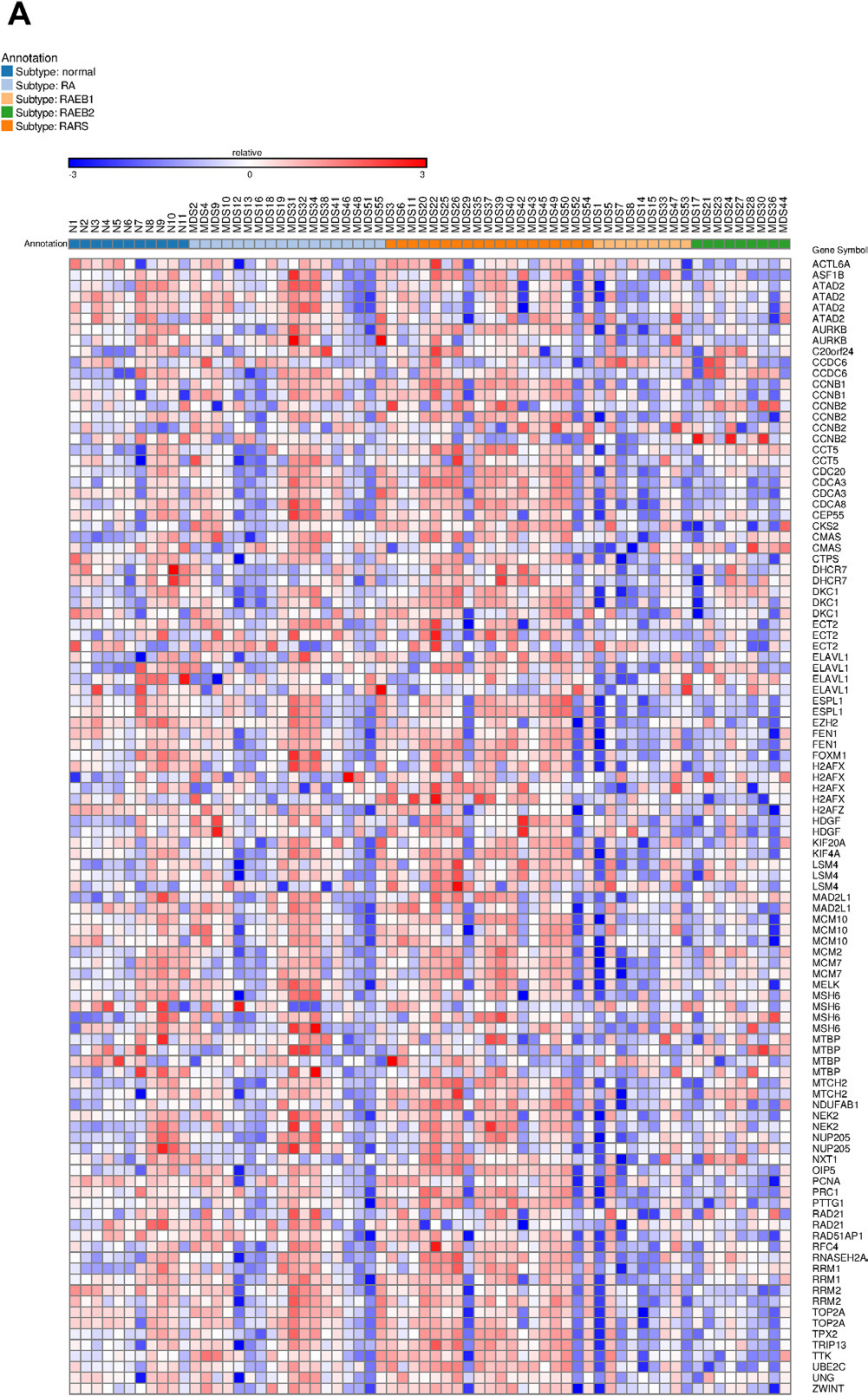

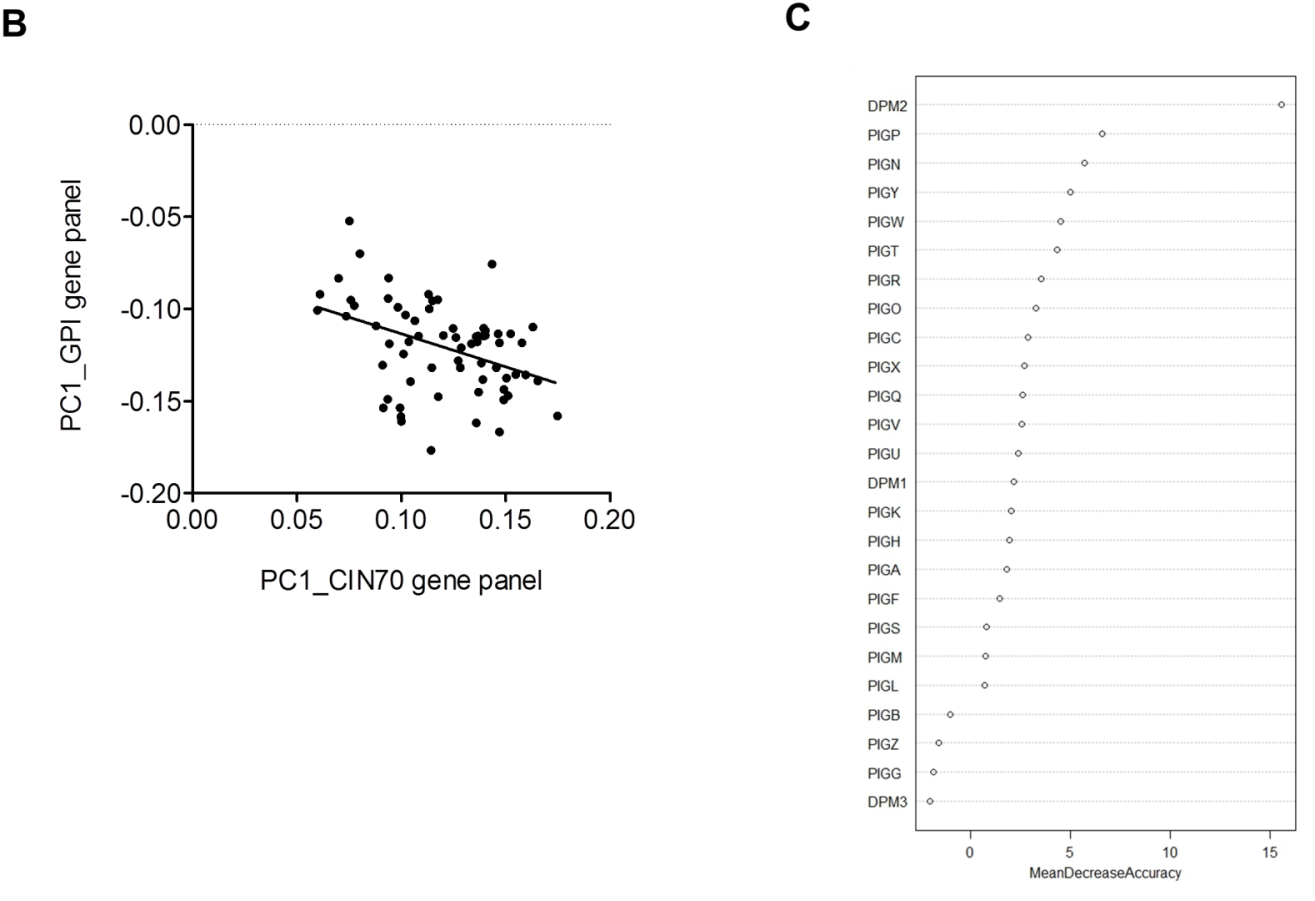
*PIGN* gene was highly ranked as a predictive biomarker of MDS risk stratification **A**. Gene expression heat map showing expression of the CIN70 signature was associated with MDS risk stratification in CD34+ cells isolated from bone marrow samples of 55 MDS patients and 11 normal controls (GSE4619) [36, 37]. **B**. 2D scatter plot showed a significantly (p = 0.0007) negative correlation (Pearson r =-0.4068) between the GPI anchor biosynthesis gene panel and the CIN70 signature by plotting the first principal component (PC1) of each individual per gene panel. **C**. *PIGN* was ranked third among GPI-AP biosynthesis genes in predicting MDS risk stratification based on a Random Forest classifier using Mean Decrease in Accuracy as predictor.

We initially analyzed array data generated from 55 MDS patients and 11 normal controls (GSE4619) [36]. The patients were sub-classified as follows: RA (18 patients), RARS (19 patients), RAEB1 and REAB2 (18 patients). Overall, CIN70 genes were expressed in a MDS disease subtype-dependent manner with a relatively lower expression in high-risk disease subtypes (REAB-1 and RAEB-2) compared to the low risk subtypes (RA and RARS) and normal controls [37]. This gene expression heat map showed that the expression of the CIN70 gene panel was associated with MDS risk stratification (Figure 1A). A randomForest analysis further demonstrated a significant (p = 0.0007) correlation (Pearson r =-0.4068) between the GPI-anchor biosynthesis gene panel and the CIN70 genomic instability marker panel (Figure 1B). Furthermore, the mean decrease in accuracy identified *PIGN* as highly important (i.e. 3^rd^ ranked) among the GPI-AP biosynthesis genes in predicting MDS progression risk (Figure 1C).

### *PIGN* gene expression aberrations occur in a subgroup of patients with MDS or AML-MRC

**Figure 2:**
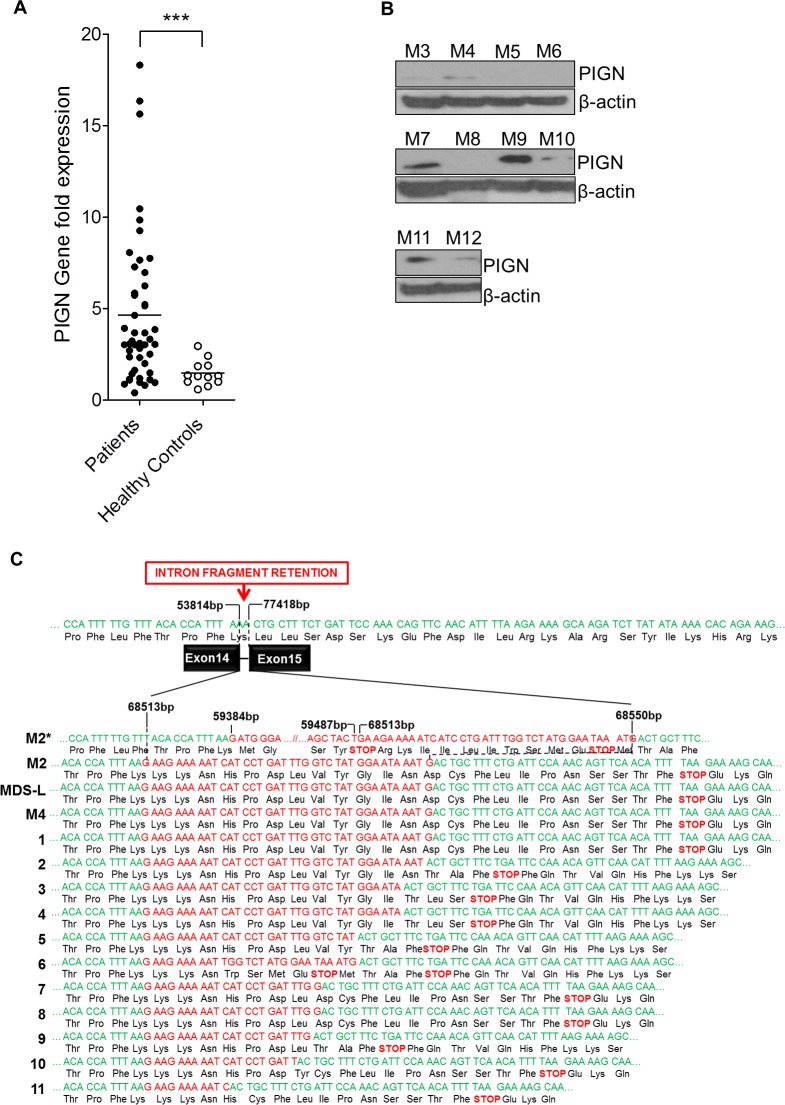
*PIGN* gene expression aberration was due to truncation **A**. RT-qPCR data on patient samples showed that a subgroup of MDS/AML-MRC patients had a significant difference (***p<0.0001) in *PIGN* gene expression than normal controls. **B**. In that same subpopulation of patients their PIGN protein expression was lost or suppressed. **C**. Sequence analyses on CD34+ cells revealed the presence of intron fragment retentions resulting from splice defects between exons 14 and 15 caused frameshifts and premature termination; samples M1, M2 and M4 were from AML patients; samples 1-11 represented the results of RNA-seq junction file data analyses from AML patients in the dbGAP study phs001027.v1.p1. Intron base positions (bp) were based on NCBI reference sequence NG_033144.1.

We used RT-qPCR to determine the *PIGN* gene expression profiles of CD34+ mononuclear cells harvested from the peripheral blood or bone marrow aspirates of 48 patient samples with either high risk MDS or AML-MRC and 12 healthy volunteers. Our results revealed that the majority (~60%) of these patients had a significantly (p<0.0001) higher expression of the *PIGN* gene in comparison with the cells from healthy normal controls (Figure 2A). Moreover, 15 of 35 patient samples examined for both *PIGN* transcription and translation had an aberrant expression pattern (i.e. increased transcriptional activity but diminished to no protein production) (Table 1 and Figure 2B). Overall, these data indicated that a subgroup of patients with high risk MDS or AML-MRC appeared to have *PIGN* expression aberration with increased gene expression but diminished protein production.

**Table 1:** *PIGN* gene and protein expression status in MDS or AML-MRC patients

ID	Age/Sex	TP53 Deletion	PIGN Protein Expression	^a^ PIGN Gene Fold Expression	Karyotype (Normal/Complex)
M1	60/F	+	-	3.787	Complex
M2	27/F	+	-	7.653	Complex
M3	87/M	-	+	1.187	Complex
M4*	59/F	-	+	3.927	Complex
M5*	59/F	-	-	2.703	Complex
M6	61/M	-	-	4.639	Complex
M7	78/M	-	+	1.636	Complex
M8	64/M	-	-	0.398	Complex
M9	29/F	-	+	N.D	Complex
M10	29/F	-	+	1.435	Complex
M11	68/M	-	+	2.002	Complex
M12	61/F	-	+	3.228	Normal
M13	63/F	+	+	0.873	Complex
M14	55/F	-	N.D	2.323	Normal
M15	67/F	-	-	5.158	Complex
M16	67/F	-	-	15.633	Complex
M17	73/F	-	N.D	1.150	Complex
M18	27/M	-	+	2.513	Complex
M19	48/F	-	-	7.756	Complex
M20	66/F	-	N.D	3.045	Complex
M21	72/F	+	-	6.974	Complex
M22	45/F	-	+	5.737	Normal
M23	46/M	-	-	3.857	Complex
M24	59/M	-	-	10.461	Complex
M25	47/F	-	N.D	6.246	Complex
M26	27/F	+	-	5.227	Complex
M27	37/F	-	N.D	18.311	Complex
M28	56/F	+	-	9.260	Complex
M29	84/F	-	N.D	3.068	Complex
M30	61/M	-	+	3.328	Normal
M31	74/M	+	N.D	5.849	Complex
M32	61/M	-	+	2.354	Complex
M33	74/M	-	N.D	16.343	Complex
M34	74/M	-	N.D	3.031	Complex
M35	65/F	-	N.D	0.808	Normal
M36	65/F	-	+	0.959	Normal
M37	81/F	+	+	1.490	Complex
M38	75/F	-	+	3.671	Normal
M39	85/M	-	N.D	3.026	Normal
M40	71/M	-	+	3.021	Complex
M41	77/M	-	+	0.962	Complex
M42	62/M	-	-	7.275	Complex
M43	49/F	-	-	9.842	Complex
M44	51/F	-	+	3.676	Normal
M45	51/F	-	N.D	1.096	Normal
M46	58/M	+	+	2.825	Complex
M47	68/F	+	+	1.113	Complex
M48	59/M	-	N.D	8.061	Complex

^a^ Mean fold difference in gene expression in patients compared to *PIGN* gene expression in normal healthy control PBMCs.

+: detected

-: not detected

N.D: no data available

Mono: mononuclear cells

*: M4 and M5 from the same patient; M4 at pre-treatment phase and M5 at relapse phase.

### *PIGN* gene expression aberrations were caused by novel intronic retention mutation between exons 14 and 15

We further explored the cause of this *PIGN* gene expression aberration by cloning and sequencing the *PIGN* transcripts from 3 patient samples (M1, M2, and M4) and a cell line (MDS-L) which had significantly high *PIGN* gene expression but no protein expression. Our results revealed the retention of aberrant short intronic fragments (i.e. 11bp to 142bp) between exons 14 and 15 (Figure 2C; 1-4). The predicted product of this mutation is a truncated protein around ~46 kDa which is less than half of the normal protein size (i.e. ~106 kDa). Interestingly, we identified similar variants of this mutation in 11 AML patients from junction files generated from the RNA-seq data of 19 AML patients (dbGaP Study Accession: phs001027.v1.p1) (Figure 2C; 1-11) [38]. Further examination at the resolution of individual bases of these aberrant transcripts revealed that these intron fragments were similar to those originally identified in the patients with *PIGN* gene expression aberrations.

### The novel intronic retention mutations are present in leukemic cells but not in non-leukemic cells and are associated with a relatively high frequency of GPI-AP deficiency

**Figure 3:**
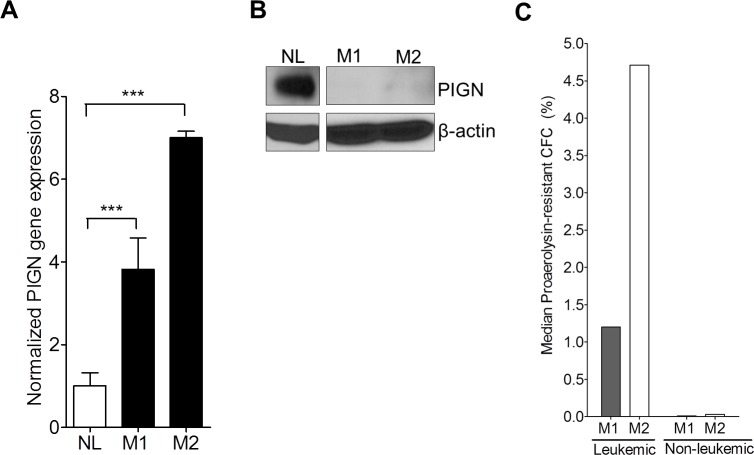
*PIGN* expression aberration resulted in an increased frequency of GPI-AP deficiency **A**. RT-qPCR showed that *PIGN* gene expression in leukemic cells from AML patients M1 and M2 were significantly (***p<0.0001) higher (i.e. 3- to 7-fold) than in normal control cells from healthy individual (NL). One way ANOVA Tukey’s post-hoc test; error bars represent standard deviation from the mean fold change in gene expression. **B**. PIGN protein expression was lost in patients M1 and M2. **C**. Frequency of GPI-AP loss was much higher in leukemic clones than in the non-leukemic clones in the respective AML patients. For detailed calculations of frequency of GPI-AP deficiency please review citation [13]. *Leukemic and non-leukemic cells were sorted using the following markers: HLA-DR, CD13, CD117 and CD45 as described earlier with some modifications [54].

Using RT-qPCR, we examined *PIGN* gene expression in sorted leukemic cells from 2 AML patients (M1 and M2). Both patients contained *TP53* gene deletion mutations (Table 1). *PIGN* gene expression in the leukemic cells from these two patients was at least 3~7-fold higher than in the non-leukemic cells (NL); but PIGN protein expression was not detectable in those leukemic cells (Figure 3A–3B). We then sub-cloned and sequenced *PIGN* transcripts from the sorted leukemic cells and non-leukemic cells. Interestingly, we observed the retention of segments (38 bp and 142 bp) of the intervening intron between exons 14 and 15 in the leukemic cells which resulted in frameshifts and led to the occurrence of premature termination codons (PTCs) (Figure 2C); but not in the non-leukemic cells.

Elevated frequency of GPI-AP deficiency has been linked with genomic instability and leukemic progression [16]. In order to explore the genetic stability status of those patients, we conducted proaerolysin-resistant colony forming cell (CFC) assays on both sorted leukemic and non-leukemic cells from patients M1 and M2. We then calculated the GPI-AP deficiency frequency of the two AML patients as previously described [13]. The median frequencies (GPI-AP deficiency frequency) of proaerolysin-resistant leukemic CFC formation for M1 and M2 were 1.20% and 4.71% (ranging from 0.27 to 3.02% and 2.88 to 6.46%) respectively; however, the median frequencies (GPI-AP deficiency frequency) of proaerolysin-resistant non-leukemic CFC formation were 0.009% and 0.029% (ranging from 0.004% to 0.013% and 0.007% to 0.075%) respectively (Figure 3C). The GPI-AP deficiency frequency in a normal population is approximately 0.002% [13]. Thus, the GPI-AP deficiency frequencies in the leukemic cells were 100 times higher than in the non-leukemic cells.

### *PIGN* gene expression aberrations occur during leukemic transformation and progression

**Figure 4:**
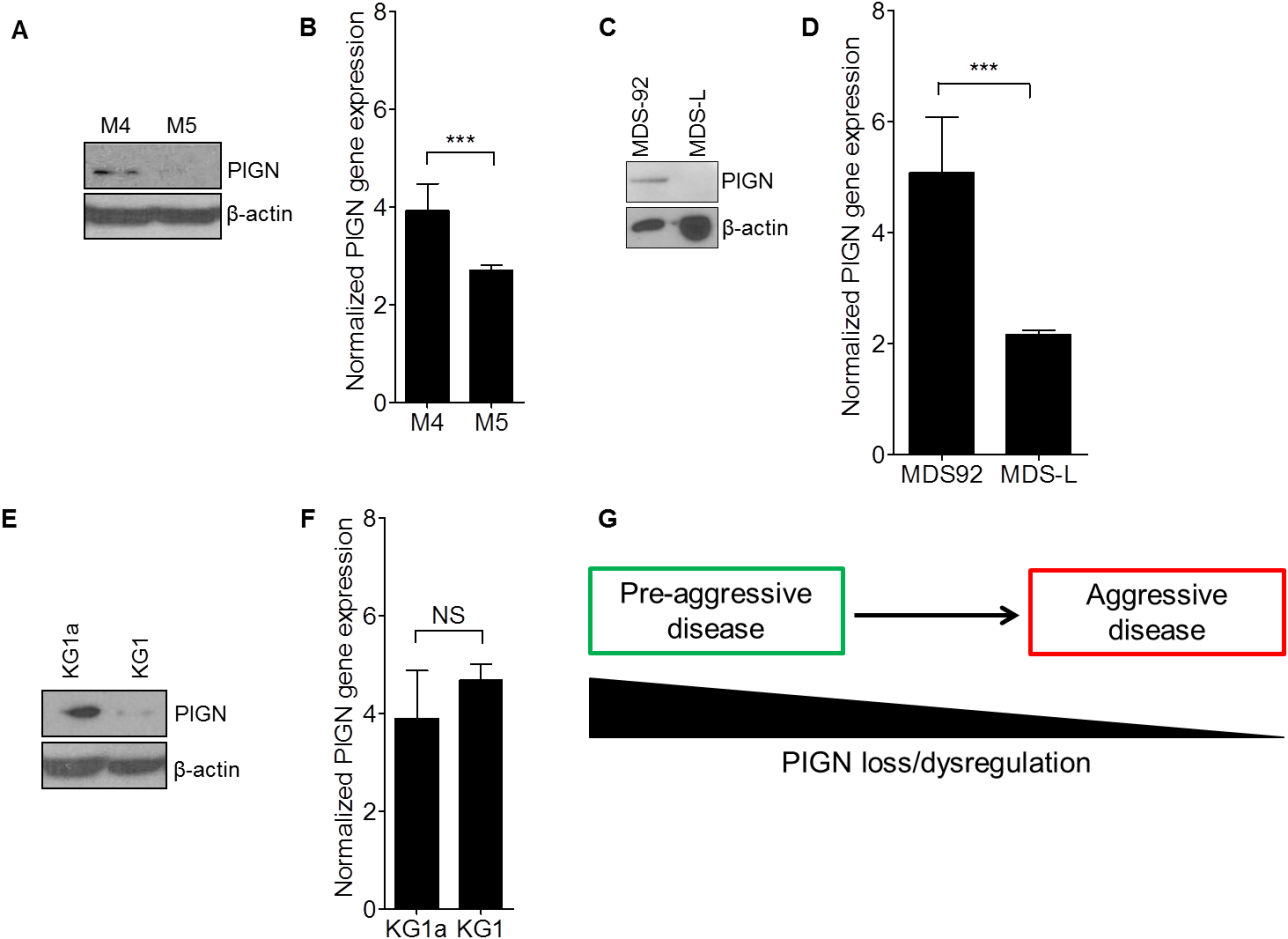
*PIGN* expression aberration was a marker of leukemic transformation and progression **A**. PIGN protein was progressively lost in an AML patient (M4 and M5) and **B**. *PIGN* gene expression was significantly (***p<0.0001) downregulated from pre-treatment (M4) to relapse (M5). Error bars represent standard deviation from the mean fold change in gene expression. **C** and **D**. MDS92 and MDS-L shared the same origin. **(C)** PIGN protein progressively lost from the MDS phase (MDS92 cells) to the leukemic phase (MDS-L cells) and **(D)** *PIGN* gene expression was significantly (***p<0.0001) higher in MDS92 cells than in MDS-L cells. **E**. Similarly, PIGN protein expression was more suppressed in the myeloblastic phase (KG1) comparing to its myeloid derivative (KG1a) but **F**. no significant (NS) difference in gene expression was observed between the KG1a and KG1cell lines. However, the *PIGN* gene transcriptions in all of the above-mentioned samples were elevated 2- to 5- fold in comparison with *PIGN* gene expression in CD34+ cells from healthy individuals. Error bars represent standard deviation from the mean fold change in gene expression. **G**. This simplified model depicted the loss of PIGN protein with disease progression from a less aggressive disease stage to a more aggressive disease stage.

Due to our initial identification of partial intron retentions in the sorted leukemic cells, we examined *PIGN* gene and protein expression in relation with disease progression in a refractory AML patient (Figure 4A–4B). That patient had 65% leukemic blasts during the pre-treatment phase (M4) and 42% leukemic blasts at the relapse phase (M5). We detected an intron fragment retention between exons 14 and 15 in the pre-treatment mononuclear cells of this patient that was similar to those intron fragment retentions earlier identified in the sorted leukemic cells in M1 and M2 (Figure 2C). However, this intron fragment was not detected in the mononuclear cells collected at the relapse phase. Furthermore, we observed *PIGN* gene expression aberrations in both phases of disease progression (M4 and M5) in this AML patient, with higher gene expression (~4-fold) in the pre-treatment phase than in the relapse phase (~2.5-fold) compared to normal healthy control cells (Figure 4A–4B), but more suppressed protein expression in the relapse phase.

In order to examine whether *PIGN* gene expression aberration occurs during leukemic transformation, we employed a cell line model of MDS transformation to AML. This model involves two cell lines (MDS92 and its blastic subline MDS-L) generated from a single patient but with distinct phenotypes representative of the MDS phase and the AML phase of leukemic progression respectively [39]. We examined *PIGN* gene and protein expression in these two cell lines. PIGN protein expression was relatively higher in MDS92 cell line but was not detected in MDS-L cell line (Figure 4C). Moreover, we observed a relatively high *PIGN* gene expression in MDS92 cells (~5.1-fold) and MDS-L cells (~2.2-fold) compared to normal non-leukemic mononuclear cells (Figure 4D). Thus, *PIGN* gene expression aberration was more obvious in the leukemic phase than in the MDS phase. Interestingly, we detected the same intron fragment retention in the leukemic phase MDS-L cell line as the one we identified in leukemic cells from M2 and M4 (Figure 2C). This mutation was however not detected in the MDS92 cells. Thus, *PIGN* expression aberration occurs during MDS leukemic transformation and progression and is marked by the presence of partial intron retention mutations between exons 14 and 15, and ultimately the progressive loss of PIGN protein expression. We also observed a similar PIGN expression aberration pattern in one (KG1) of two leukemia cell lines (KG1 and KG1a) originated from a single patient, KG1 harboring a myeloblast phenotype but KG1a bearing a stem/progenitor-like phenotype (Figure 4E–4F). However, no intron fragment retention was detected in either cell lines while *PIGN* gene expression was only marginally different between these two cell lines. Overall, the progressive loss of PIGN protein expression in these leukemic cells and cell lines in the different MDS/leukemic progression phases indicated that PIGN loss may mark myeloid leukemia progression from a less aggressive disease state to a more aggressive one (Figure 4G). However, the partial intron retention mutations between exons 14 and 15 only occur in a subgroup of patients, especially those patients who have not received chemotherapy yet.

### The genomic instability status in leukemic cells was driven by *PIGN* gene expression aberration and was TP53 regulatory pathway independent

**Figure 5:**
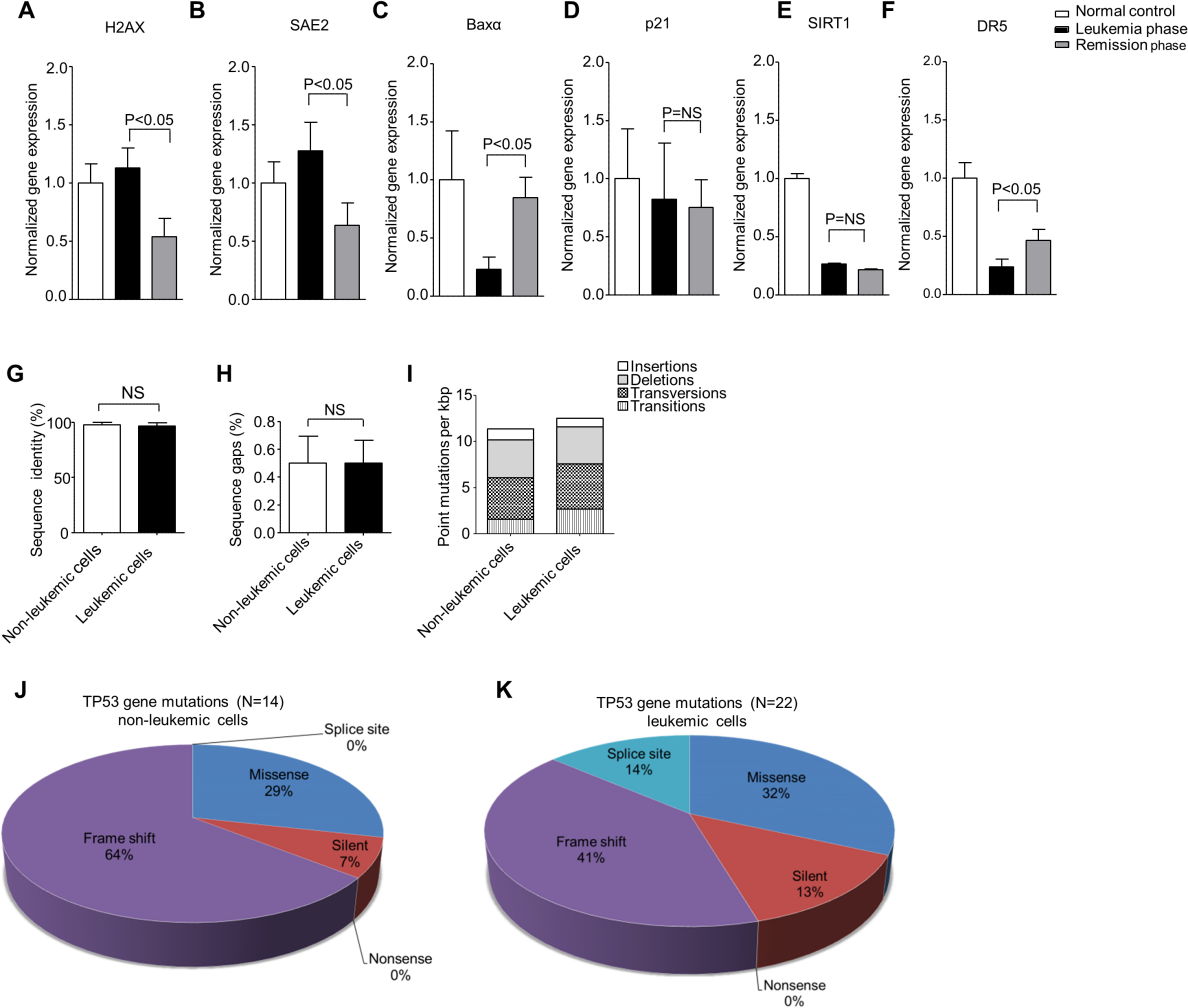
*PIGN* expression aberration was associated with genomic instability in leukemic cells and was TP53-pathway independent **A** and **B**. *TP53*-independent genomic instability/DNA damage markers (*H2AX* and *SAE2*) gene expression were significantly (p<0.05) upregulated in the leukemic phase compared to remission phase. **C**. The expression of *TP53*-targeted apoptosis marker *BAXα* was downregulated in both leukemic phase and remission phase though it was more significantly (p<0.05) in the PMNC rich with leukemic cells. **D**. The *TP53* target gene involved in cell cycle control (*p21*) was not significantly (NS) different between the active leukemia and remission phase and could point to a TP53-independent mechanism. **E**. The *TP53* deacetylase and deactivator, *SIRT1* was also not significantly (NS) different between the leukemic and remission phase of disease progression. **F**. The expression of the TP53 target, *TRAIL death receptor 5 (DR5)* was significantly downregulated in the leukemic cell rich active leukemia phase compared to the remission phase but *DR5* expression was below 50% of the normal control in both the leukemia and remission phases. Genomic instability biomarkers not regulated by TP53 (*H2AX* and *SAE2*) showed significantly transcriptional activation in mononuclear cells rich with leukemic cells in the active leukemia phase but not in mononuclear cells in the remission phase. Results were analyzed using a One-way ANOVA followed by Tukey’s post hoc tests. P-values <0.05 were considered statistically significant. **G** and **H**. the PBMCs from patient M2 were sorted into leukemic and non-leukemic cell populations followed by Sanger sequencing of approximately 2,300 bp of DNA encoding for intron and exon regions ranging from exons 2-11 of the *TP53* gene revealed no significant (NS) difference in the overall sequence identity (%) of sequenced **(G)** introns and **(H)** exons between the non-leukemic and the leukemic cells with reference to the *TP53* gene sequence (NC_000017.9). **I**. Combined intron and exon mutation frequency normalized to kilo bp (kbp) of the *TP53* gene in non-leukemic and leukemic cells in patient M2. Qualitative analysis of sequence alterations of *TP53* gene coding sequences (exons) of **J**. non-leukemic cells and **K**. leukemic cell populations in patient M2 showed no significant difference. Statistical differences were analyzed using Student’s t-test. P-values ≤0.05 were considered statistically significant. ‘NS’ indicates statistically non-significant (p>0.05) differences.

We further investigated the role of *PIGN* gene expression aberration in genomic instability by comparing the gene expression levels of a group of genomic instability/DNA damage related biomarkers in peripheral blood mononuclear cells collected from patient M2 at leukemia active phase and leukemia remission phase. We observed that the biomarkers not regulated by *TP53* (*H2AX* and *SAE2*) manifested a significant transcriptional activation in the leukemia active phase but not in the remission phase (Figure 5A–5B). *H2AX* is a genomic instability suppressor gene and *SAE2* encodes a protein involved in double strand DNA break repair [40, 41]. *BAXα*, a pro-apoptotic gene, was significantly downregulated in the leukemia active phase as well (Figure 5C). However, the expression of TP53 target gene *p21* and the *TP53* deacetylase gene *SIRT1* was not significantly different between leukemia phase and remission phase (Figure 5D–5E). The *TP53*-dependent TRAIL death receptor *DR5* was upregulated in the remission phase but was still about 50% below the *DR5* gene expression in the normal control (Figure 5F).

*TP53* gene deletion was observed in both M2’s leukemic cells and non-leukemic cells. Approximately 2,300 bp of DNA from non-leukemic and leukemic cells spanning exons 2-11 of the *TP53* gene was analyzed by Sanger sequencing. However, only three sequence alterations could be verified as a conserved deletion or missense mutations between the different cell types (Table 2). We found no significant difference in sequence identity (%) between non-leukemic and leukemic cells derived from patient M2 (Figure 5G–5H). The overall mutation rate was also similar between non-leukemic and leukemic cells with 11.4/kbp and 12.5/kbp respectively (Figure 5I). Non-leukemic cells displayed a total of 14 sequence alterations in the coding sequence whereas leukemic cells displayed 22 (Figure 5J–5K). However, *PIGN* gene expression aberration was only observed in the leukemic cells. Thus, we proposed that *PIGN* gene expression aberration may be the driving force of high genomic instability in the leukemic cells.

**Table 2:** Verified common non-leukemic and leukemic mutations in the TP53 gene

Position^a^	Exon	Nucleotide change	Type	AA Change	SIFT^b^
11,031	2	G>A	Non-synonymous	p.S9N	neutral
11,470	4	C>G	Non-synonymous	p.P80R	neutral
12,379	5	C>-	Frameshift deletion	p.N131fs	NA

^a^ Position in GenBank NC_000017.9

^b^ http://p53.iarc.fr/TP53GeneVariations.aspx

**Figure 6:**
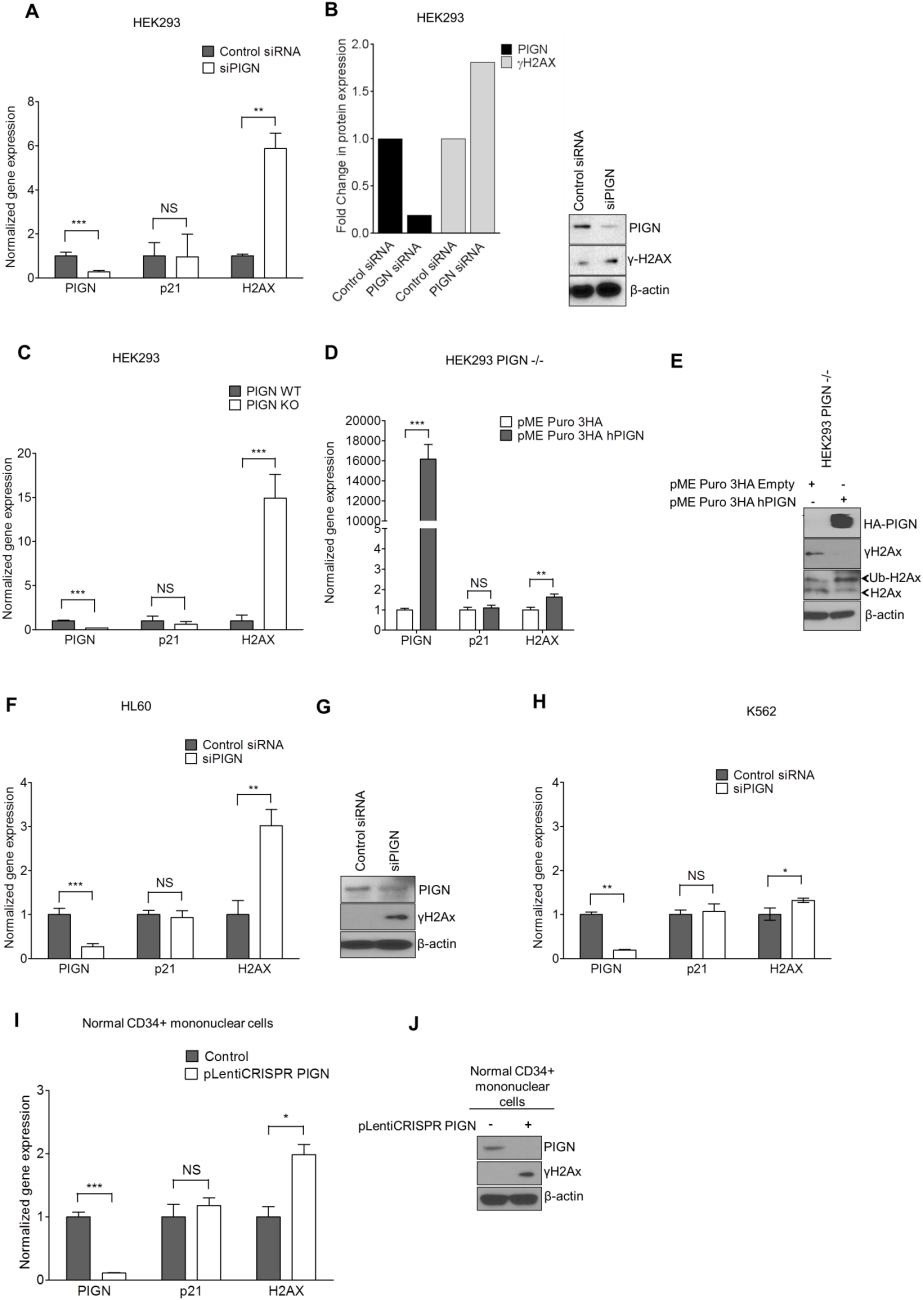
*PIGN* gene expression suppression was associated with genomic instability; and reintroduction of PIGN gene expression restored genomic stability in a TP53-pathway independent manner **A**. *PIGN* suppression (***p=0.0008) in HEK293 (TP53wt) cells resulted in the upregulated gene expression of *H2AX* (**p=0.0029) but no significant change (NS) in *p21* gene expression. **B**. *PIGN* suppression resulted in DNA damage response via a ~50% upregulation of γH2AX transcription and translation in HEK293 cells. **C**. *PIGN* deletion (***p< 0.0001) results in a ~15 fold increase (***p=0.0003) in *H2AX* gene expression but a marginal increase in *TP53*-dependent *p21* gene expression in HEK293 cells. **D**. Restoration of *PIGN* in PIGN null (CRISPR/Cas9 deletion) HEK293 cells via transfection of *PIGN* expression plasmid results in a marked upregulation (i.e. ~400-fold) in *PIGN* gene expression with a ~1.6-fold increase (**p= 0.0056) of *H2AX* transcription and no significant (NS) change in *p21* gene expression. **E**. Restoration of *PIGN* expression in PIGN null HEK293 cells ameliorates genomic instability as indicated by *γH2AX* suppression while increasing the mono-ubiquitination of *H2AX* which is critical in the initiation of DNA damage response. **F** and **G**. *PIGN* loss (***p= 0.0003) in HL60 cells (TP53 null) results in a significant (**p=0.0013) upregulation of *H2AX* in both **(F)** transcription level and **(G)** translation level but not (NS) *p21* gene transcription. **H**. *PIGN* suppression (**p=0.0019) resulted in a marginal increase (*p=0.0387) in *H2AX* gene expression (NS) but not *p21* gene expression (NS) in K562 cells (TP53 inactivation mutation). Above-mentioned data indicated that *PIGN* suppression/elimination caused genomic instability was independent from *TP53*-pathway regulation. **I**. CRISPR/Cas9 ablation (***p<0.0001) of *PIGN* in normal healthy donor CD34+ mononuclear cells results in a significant (*p=0.0261) upregulation in *H2AX* transcriptional activation without a significant (NS) increase in *p21* transcriptional activation. **J**. *PIGN* loss via CRISPR/Cas9 ablation induces upregulation of γH2AX translation in normal healthy donor CD34+ mononuclear cells.

In order to further test our hypothesis that *PIGN* gene expression aberration can contribute to genomic instability regardless of *TP53* gene status, we employed the transient knockdown of *PIGN* in multiple cell lines with various *TP53* gene mutation statuses. We investigated the impact of *PIGN* gene expression suppression or silencing on the gene expression of *p21* and *H2AX* in HL60 (*TP53* deletion), K652 (*TP53* mutation), HEK293 and HEK293 PIGN KO (*TP53* wild type) cell lines and CD34+ mononuclear cells from a healthy individual. We observed that *PIGN* suppression in HEK293 cells resulted in the upregulation of *H2AX* transcription and *γH2AX* induction (Figure 6A–6B). Moreover, CRISPR/Cas9 knockout of *PIGN* in HEK293 cells confirmed a functional link between PIGN loss and the induction of genomic instability in cells and involved an increased transcription (~15-fold) of *H2AX* (Figure 6C). However, genomic instability was reduced as shown by γH2AX downregulation with the restoration of PIGN expression (Figure 6E). We confirmed these findings in K562 and HL60 cell lines (Figure 6F–6H) and in CD34+ mononuclear cells from a healthy individual (Figure 6I–6J). Interestingly, *H2AX* expression in these cell lines was not influenced by their *TP53* gene mutation status, and *p21* gene expression was not influenced by *PIGN* gene expression status. Thus, *PIGN* loss or suppression induced genomic instability in a *TP53* pathway-independent manner.

### PIGN maintains genomic stability, especially chromosomal stability, by regulating the mitotic spindle assembly checkpoint protein MAD1

**Figure 7:**
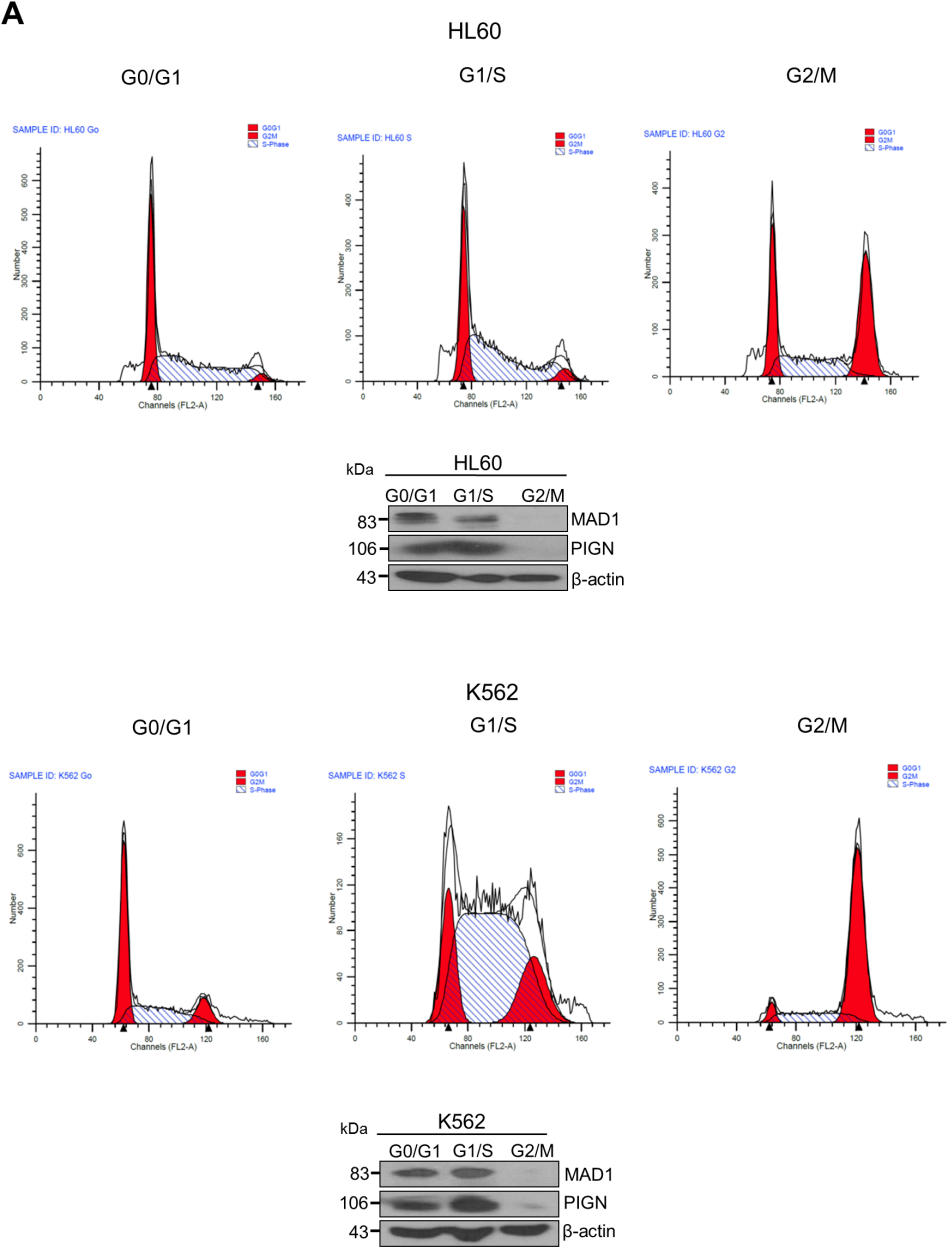

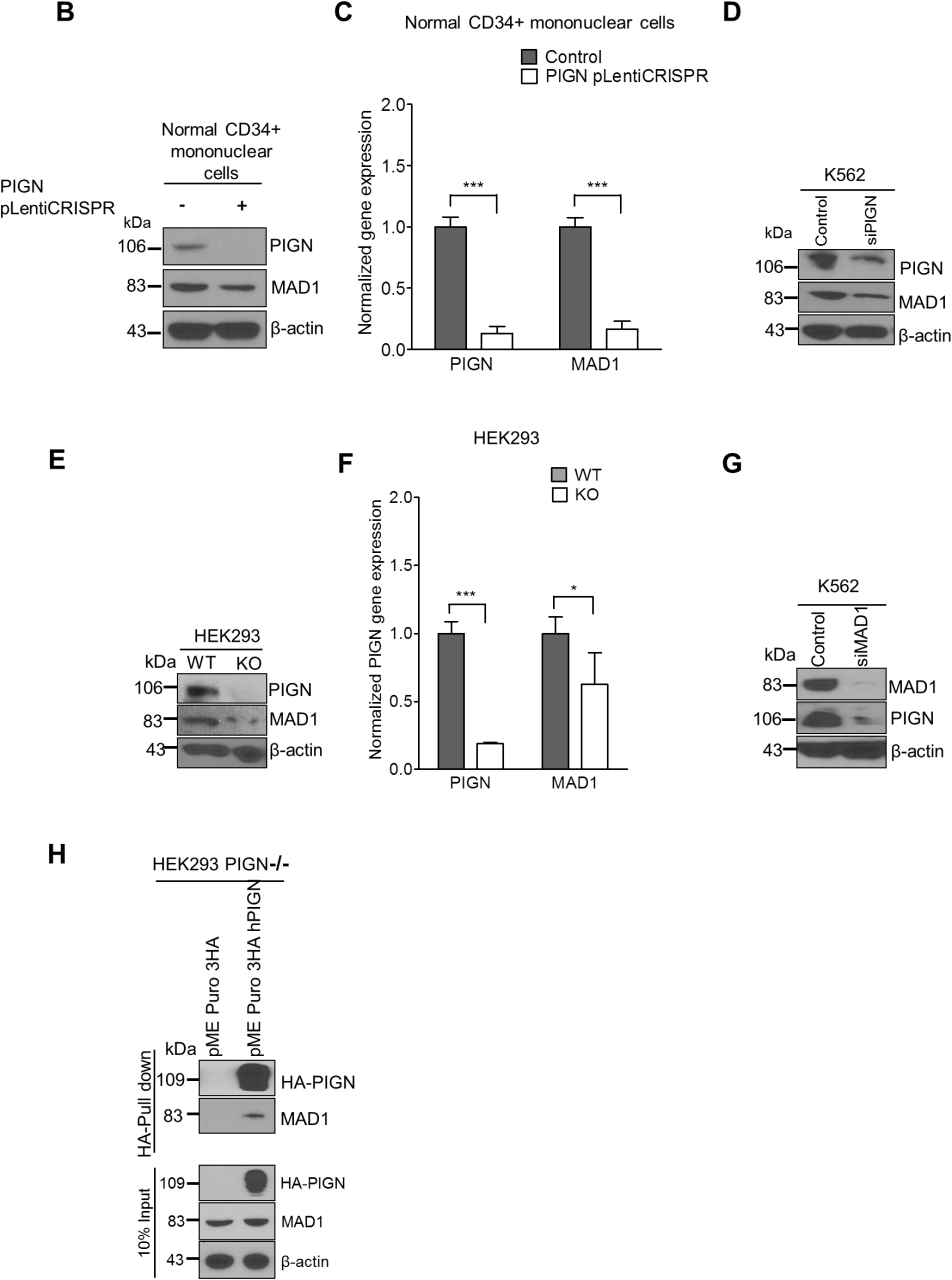

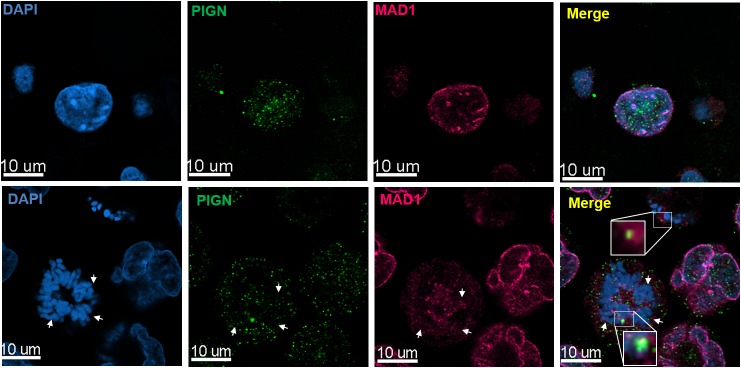

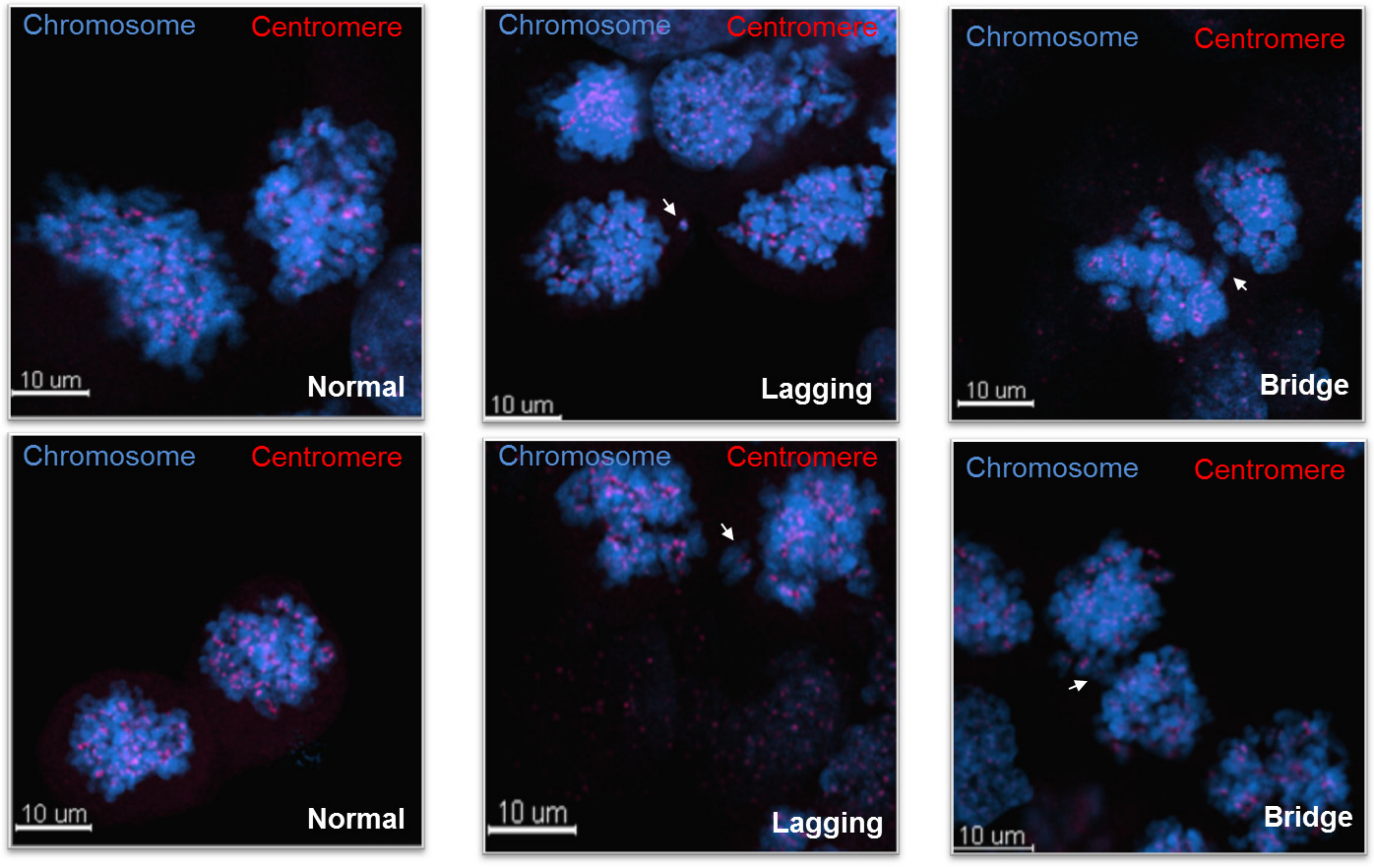

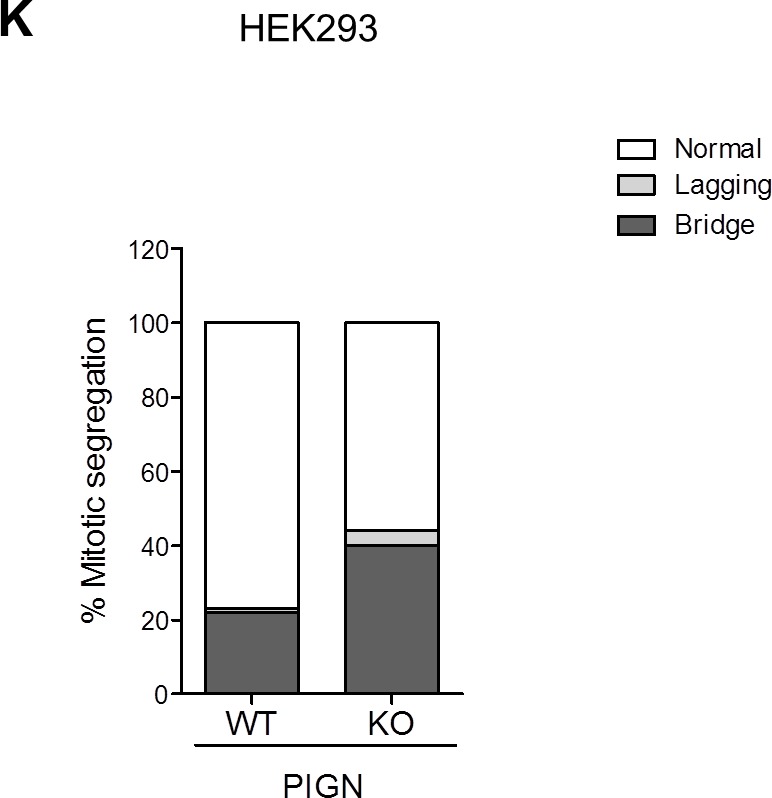
*PIGN* loss induced chromosomal instability via dysregulation of the spindle assembly checkpoint protein MAD1 **A**. PIGN and MAD1 were similarly expressed in a cell cycle-dependent manner with suppressed expression in the G2/M phase in HL60 and K562 cells. **B**. PIGN loss via CRISPR/Cas9 ablation resulted in MAD1 downregulation in normal healthy donor CD34+ mononuclear cells. **C**. *MAD1* gene expression was significantly (***p=0.0007) impacted by *PIGN* gene loss in normal healthy donor CD34+ mononuclear cells. **D**. RNAi-mediated *PIGN* suppression resulted in MAD1 downregulation in K562 cells. **E**-**F**. Comparing *PIGN* wild-type (WT) HEK293 cells and *PIGN* null (KO) HEK293 cells: *PIGN* loss is associated with downregulation of MAD1 protein expression and repression (*p=0.0509) of *MAD1* gene transcriptional activation. **G**. *MAD1* suppression was accompanied by a corresponding decrease in PIGN protein expression in K562 cells. **H**. MAD1 directly interacted with PIGN. MAD-1 was co-purified with PIGN in a HA-tag pulldown assay in *PIGN* null HEK293 cells. Input represents 10% of total protein lysate used in the HA pull down assay. **I**. Immunofluorescence image of K562 cells showed that PIGN (green) and MAD1(red) had a similar pattern of localization during the mitotic phase and co-localized (yellow) during late prometaphase. White arrows indicate groups of chromosomes in prometaphase. Upper panel: Asynchronous cells; Lower panel: Late prometaphase cells. **J-K**. *PIGN* loss in HEK293 cells results in phenotypes associated with chromosomal instability (increased lagging chromosomes and anaphase bridges). **J**. Representative images of missegregation errors observed in HEK293 cells; Blue (chromosomes) and red (centromere). White arrows indicate lagging chromosomes and positions of anaphase bridges. **K**. Quantitative analyses of missegregation errors were calculated by counting the numbers of lagging and anaphase bridges observed in a total of 100 cells randomly selected from multiple fields of view.

We sought to further investigate the mechanistic role of *PIGN* in maintaining genomic stability. We conducted cell cycle experiments by blocking cell cycle progression at G0/G1, S and G2/M phases in HL60 and K562 cells via serum starvation, double-thymidine and nocodazole treatment respectively. We observed a cell-cycle dependent expression of PIGN which correlated with the expression of the spindle assembly checkpoint (SAC) protein MAD1. PIGN and MAD1 were least expressed in the G2/M phase of the cell cycle (Figure 7A). The SAC is primarily responsible for ensuring proper chromosomal segregation during metaphase-anaphase transition [42]. We observed that *PIGN* suppression/knockout caused *MAD1* suppression, even in CD34+ mononuclear cells derived from a healthy individual (Figure 7B–7F). Alternatively, MAD1 suppression resulted in decreased expression of PIGN (Figure 7G). These findings revealed a novel reciprocal regulation between the SAC component MAD1 and PIGN. To further investigate the relationship between PIGN and MAD1, we transfected CRISPR/Cas9 PIGN KO HEK293 cells with an HA-tagged *PIGN* and performed a HA-tag pulldown assay. We observed a direct interaction between PIGN and MAD1 with the highest interaction at 48 hours post-transfection (Figure 7H). Confocal analyses also revealed co-localization of MAD1 and PIGN during prometaphase in K562 cells (Figure 7I). PIGN loss was also accompanied by an increase in the frequency of missegregation errors in *PIGN* CRISPR/Cas9 knockout HEK293 cells (Figure 7J–7K). The same experiments were conducted on a leukemia patient sample (M4), HL60 and K562 cells with similar observations (data not shown). The above data indicated that PIGN maintains chromosomal stability by interaction with the SAC protein MAD1 during the cell cycle.

## DISCUSSION

Genomic instability is a driving force for cancer initiation and progression. Previous studies have indicated that cell lines with genomic instability (i.e. Fanconi anemia and colon cancer cells with a mutator phenotype) had a marked increase in frequency of acquiring GPI-AP deficiency [14, 15, 17]. Our laboratory observations have also shown that MDS and myeloproliferative diseases (MPD) patients bearing high frequency of GPI-AP deficiency posed a higher risk for leukemic transformation [12]. Using bioinformatics tools to screen existing databases we identified the *PIGN* gene as a predictor of MDS progression risk [36]. We then observed a unique gene expression aberration pattern within a subgroup of patients with MDS or AML-MRC. We were able to link PIGN protein loss to the presence of partial retentions of the intervening intron between exons 14 and 15 that resulted in frameshifts and early translational termination. We confirmed the presence of this intron retention mutation in multiple AML patients based on RNA-seq data analyses (phs001027.v1.p1) that verified the conserved nature of this mutation. However, no such natural alternative splice variants of *PIGN* gene have been reported (Ensembl release 85: ENSG00000197563). Thus, the novel intron fragment retention identified in our study is not a natural alternative splice variant. Similar deleterious splice defects were previously reported for genes including *DMD, C9orf72* and *GR* associated with muscular dystrophy, amyotrophic lateral sclerosis/frontotemporal dementia and small cell lung cancer respectively [43–45]. Moreover, splicing factor genes have been reported to be mutated in MDS and AML [46].

We also observed *PIGN* gene expression aberration and partial intron retention in a pre-treatment sample (M4) from an AML patient. Despite progressive loss of PIGN protein expression in the relapse sample (M5), this intron fragment retention was not detected. We could not explain why partial intron retention was not detected in M5, though M5 was in the state of *PIGN* gene expression aberration. We hypothesize that chemotherapy may eliminated the clone harboring *PIGN* partial intron retention but the chemo-resistant clones survived and proliferated, which could explain why PIGN protein expression was not observed in M5. Earlier studies have demonstrated the occurrence of multiple clones with varied sensitivity to chemotherapy [47, 48].

The data from our patient samples, cell lines and existing databases are in line with previous observations that elevated frequency of GPI-AP deficiency is a marker of genomic instability and might predict a risk of leukemic transformation and progression [15–17]. Furthermore, this study indicated that *PIGN* gene expression aberration may be the key factor linking GPI-AP deficiency with CIN and leukemogenesis. Previous studies demonstrated that, unlike *PIGA*, *PIGN* gene loss would not completely eliminate GPI-AP biosynthesis [19, 21]. This piece of data may explain why the leukemic cells from patients M1 and M2 still showed sign of CFC formation reduction in proaerolysin-containing medium though the CFC counts were significantly higher than the normal control.

We further explored the role of *PIGN* gene expression aberration in genomic instability/leukemic progression and the role of the *TP53* signaling pathway in the regulation of genomic instability during leukemia progression by studying the leukemic cells from patient M2, and several cell lines. In this patient, *TP53* gene deletion was observed in both leukemic cells and non-leukemic cells and manifested with a similar mutation profile, however, the *PIGN* gene expression aberration only occurred in the leukemic cells. We found that the gene expression of *TP53*-independent genomic instability/DNA damage markers (*H2AX* and *SAE2*) were upregulated and the expression of the pro-apoptosis marker *BAXα* was downregulated specifically in the leukemic cell-rich mononuclear cells at the active leukemia phase when compared to the cells from the remission phase. However, the expressions of *TP53*-dependent biomarkers, such as *p21* and *SIRT1*, were not significantly different between the active phase and the remission phase. Furthermore, the expression of the *TRAIL death receptor* 5 (*DR5*) was below 50% of that of the normal control in both active phase and the remission phase. We further observed that suppression or elimination of *PIGN* gene expression in several cell lines and CD34+ mononuclear cells from healthy individuals induced a similar *TP53* independent pattern of genomic instability which could be reversed via *PIGN* gene expression restoration (Figure 6A–6J).

Our data demonstrated that *PIGN* gene expression aberration was associated with genomic instability in leukemic cells and was independent of the *TP53* regulatory pathway. A similar phenomenon was reported in the normal epithelium of benign breast tissue within the same breast cancer patients [49]. Wong *et al*. also previously reported the presence of functional *TP53* mutations in mononuclear cells isolated from healthy individuals [50]. It was suggested that *TP53* loss may be permissive rather than causative with regards to genomic instability [18, 51]. Thus, this is likely a reflection of the loss of the CIN suppressor PIGN, facilitating *TP53* gene loss of heterozygosity (LOH) in leukemic cells and corroborates the fact that *TP53* loss alone is insufficient for the promotion of genomic instability in those cells [18, 51, 52]. Our findings are also consistent with previous observations in Li-Fraumeni Syndrome patients and may explain why those patients are prone to develop therapy-related MDS with complex cytogenetics and poor prognosis [53].

PIGN protein was historically known as a membrane protein involved in GPI-AP biosynthesis, however, we showed that PIGN could directly interact with the SAC protein MAD1. PIGN loss resulted in the dysregulation of MAD1 during cell cycle progression and was associated with an increased frequency of mitotic missegregation. We demonstrated that PIGN and MAD1 were expressed similarly in a cell cycle-dependent manner with a subtle co-localization during prometaphase and the least expression in the mitotic phase relative to the G0/G1 and S phases. This decline in expression at mitotic block may be the natural process for microtubule/spindle detachment from the kinetochore or could be due to the spindle disrupting effect of nocodazole treatment which may in turn result in the destabilization and degradation of the MAD1-PIGN complex. This study showed for the first time that PIGN could directly interact with SAC protein complex at the mitotic phase of cell cycle to regulate chromosome stability. Thus, we postulate a novel model of PIGN regulation of chromosome stability via interaction and regulation of the SAC protein MAD1.

In conclusion, PIGN is a novel biomarker of CIN and leukemic transformation/progression in a subgroup of patients with MDS or AML-MRC. This study provides additional evidence for the necessity of updating our MDS/AML risk estimation stratification system, and may help to develop novel MDS/AML therapy specifically targeting CIN.

## MATERIALS AND METHODS

A brief description of research methods is below. Please read the Supplementary Material for detailed methodology.

### Leukemic blasts cell sorting

Leukemic blasts were sorted from CD34+ cells under BSL-2 conditions with a 16-color BD FACSAria SORP high speed cell sorter (Becton Dickinson) using the following markers: HLA-DR, CD13, CD117 and CD45 as previously described with few modifications [54].

### Selection of proaerolysin-resistant CFCs and GPI-AP deficiency frequency analysis

The selection of proaerolysin-resistant colony forming cells (CFCs) was conducted as previously described with some modifications [13].

### PIGN knockdown and CRISPR/Cas9 knockout studies

RNAi-mediated *PIGN* knockdown experiments were conducted using the Nucleofector™ II Device (Amaxa) in conjunction with the Cell line Nucleofector™ Kit V reagent kit (Amaxa). CRISPR/Cas9 experiments were conducted according to a modified LentiCRISPRv2 (Addgene plasmid #49535) protocol [55]. The gRNA (AAACGGTCATGTAGCTCTGATAGC) we employed targets *PIGN* at exon 4 and results in a frameshift [21].

### *TP53* sequence analyses

One microgram of DNA isolated from non-leukemic cells and leukemic cells was amplified using primers covering exons 2-11 of the *TP53* gene including intron/exon boundaries according to instructions in the IARC database (http://p53.iarc.fr) [56]. Seven PCR reactions per sample (non-leukemic and leukemic) were spin column purified and sequenced using a 3130XL capillary sequencer (ABI systems) with the same primers in both reverse and forward directions. The obtained sequence was analyzed using the software FinchTV version 1.4.0 and nucleotide BLAST (http://blast.ncbi.nlm.nih.gov/Blast). Sequencing products were aligned to the *TP53* GenBank sequence NC_000017.9 and sequence alterations were identified partially through manual inspection. Sequence alterations were correlated with the coding sequence of the TP53 protein and the impact on protein Sorting Intolerant From Tolerant (SIFT) was determined using the IARC database (http://p53.iarc.fr/p53Sequence.aspx) [56, 57].

### Bioinformatics analyses and statistical analyses

The GENE-E (http://www.broadinstitute.org/cancer/software/GENE-E/) matrix visualization and analysis platform was used to generate a heat map of the CIN70 signature of the CD34+ cells of 55 MDS patients and 11 healthy controls utilizing data generated on the Affymetrix GeneChip U133 Plus2.0 platform from the study GSE4619 [36, 37]. The randomForest v4.6-12 R package with default parameters was used in a randomForest analysis to classify patients based on MDS risk stratification. RNA-seq Analysis study was conducted on raw RNA-seq files from the dbGAP study phs001027.v1.p1. For details, please read the Supplementary Data.

MDS92 and MDS-L cells were a gift from Dr. Kaoru Tohyama, Department of Laboratory Medicine, Kawasaki Medical School Kurashiki, Okayama, Japan. HEK293 CRISPR KO cells and pME PURO 3HA hPIGN expression plasmid were the gifts from Drs. Taroh Kinoshita and Yoshiko Murakami, Research Institute for Microbial diseases, Osaka University.

## SUPPLEMENTARY MATERIALS FIGURES AND TABLES


