# Supplementary material for: PIGN gene expression aberration is associated with genomic instability and leukemic progression in acute myeloid leukemia with myelodysplastic features
Source: Oncotarget. 2017 Feb 7;8(18):29887–905. doi: 10.18632/oncotarget.15136 (PMC5444711; doi:10.18632/oncotarget.15136)
Supplement: Supplementary file 1 [file oncotarget-08-29887-s001.pdf]

# *PIGN* gene expression aberration is associated with genomic instability and leukemic progression in acute myeloid leukemia with myelodysplastic features

## SUPPLEMENTARY DATA

## MATERIALS AND METHODS

### Patient selection

All patients in this study were evaluated at the Milton S. Hershey Cancer Institute at the Pennsylvania State University College of Medicine between January 2013 and August 2016. Informed consent was obtained from each patient prior to obtaining peripheral blood and bone marrow aspirates in accordance with an approved IRB protocol (No. 40969). MDS and AML patients were diagnosed according to WHO guidelines [1, 2].

### Cell lines

KG1, KG1a and K562 cells (ATCC) were cultured in IMDM supplemented with 20% FBS. MDS92 and MDS-L cells were cultured in RPMI-1640 supplemented with 10% FBS, 50ng/ml IL-3 (Miltenyi Biotec) and 50µM 2-mercaptoethanol (Fisher Scientific). HEK293 and HEK293 PIGN CRISPR/Cas9 knockout cells were cultured in DMEM supplemented with 10% FBS. Cell lines were passaged on average every 3-4 days. CD34+ mononuclear cells were cultured in DMEM/F12 supplemented with 10% FBS, 50µM 2-mercaptoethanol, Glutamax (Life Technologies), MEM non-essential amino acids (Life Technologies) and StemMACS HSC expansion cocktail (Miltenyi Biotec). All cells were cultured at 37°C under 5% CO2 conditions.

### Isolation of mononuclear cells and CD34+ cells

Mononuclear cells were isolated from the blood or bone marrow aspirates of donors using the Ficoll-Paque PLUS reagent (GE Healthcare) as earlier described [3]. Where applicable, peripheral blood or bone marrow mononuclear cells were used for CD34+ mononuclear cells isolation via magnetic-activated cell sorting (MACS) in conjunction with anti-CD34 microbeads (Miltenyi Biotec) according to the manufacturer's protocol.

### Leukemic blasts cell sorting

Leukemic blast sorting was conducted under BSL-2 conditions with a 16-color BD FACSAria SORP high speed cell sorter (Becton Dickinson) in the Institutional Flow Cytometry Core Facility. Leukemia and non-leukemia cells were sorted using the following markers HLA-DR, CD13, CD117 and CD45 as earlier described with few modifications [4]. CD34+ mononuclear cells that expressed HLA-DR, CD13, CD117 and CD45 were sorted as leukemia cells; the rest of the CD34+ mononuclear cells were sorted as non-leukemia cells.

### Selection of proaerolysin-resistant CFCs and GPI-AP deficiency frequency analysis

Bone marrow CD34+ mononuclear cells were processed for growth of colony forming units (CFCs). The bone marrow CD34+ mononuclear cells were cultured in 1ml Methcult complete medium w/o Epo (Miltenyi Biotec) with or without 1 nM of proaerolysin. The selection of proaerolysin-resistant colony forming cells (CFCs) was conducted as earlier described with some modifications [3]. The frequency of GPI-AP deficiency was calculated as previously described [3].

### Gene expression analyses

Total RNA was isolated using the RNeasy Mini Kit (Qiagen), quantified using the NanoDrop 1000 (Thermo Scientific) and reverse transcribed using the High Capacity cDNA reverse transcription kit (Life Technologies) on the Mastercycler® Nexus (Eppendorf). Quantitative real-time (PCR) qPCR step was conducted on the StepOnePlus™ real time PCR System (ABI systems) using the following gene expression assays (Life Technologies): *PIGN* (Hs00202443_m1), *MAD1*(Hs00269119_m1) and 18S rRNA (Hs99999901_s1) gene expression as internal reference control. The *PIGN* gene expression assay was designed to have the best coverage for the reported transcript variants of the *PIGN* gene and targets the exon boundary between exon 5 and exon 6. *H2AX*, *Baxα*, *DR5*, *SIRT1*, *SAE2* and *p21* primers as well as *GAPDH* and *HPRT* internal control primers (Integrated DNA Technologies) (see Supplementary materials for primer sequences) were used with Power SYBR® Green PCR Master Mix (Life Technologies). The selected gene expression profile in the leukemic phase and non-leukemic phase were conducted via reverse transcription–qPCR (RT-qPCR) as earlier described [5]. For all experiments,

samples were run in triplicates and expression data was normalized to *PIGN* gene expression in normal healthy control peripheral blood mononuclear cells or CD34+ mononuclear cells. Gene expression was calculated using the delta delta Ct method.

### PIGN sequence analyses

RT-PCR was conducted on SuperScript One step RT-PCR system with Platinum Taq kit (Invitrogen) using total RNA as the template. The PCR products were cloned into PCR2.1 TOPO vector (Invitrogen) and clones were selected on Amp +X-gal blue/white selection plates. The plasmids were isolated by the GeneJET Plasmid Miniprep Kit (Life Technologies). M13 universal primers (Life Technologies) were used for screening the PCR insert size and sequencing was conducted at Molecular Cloning Laboratories (MCLAB) at South San Francisco, CA. Sequencing data was analyzed with reference to *PIGN* NCBI Reference Sequences: NG_033144.1 and NM_012327.5 using the CLC Sequence Viewer Version 7.6 (QIAGEN Aarhus A/S) in conjunction with manual inspection. Impact of aberrations on protein coding was conducted using the Sequence Manipulation Suite and Polyphen-2 [6, 7].

### PIGN knockdown and CRISPR/Cas9 knockout studies

RNAi-mediated *PIGN* knockdown experiments were conducted using the Nucleofector™ II Device (Amaxa) in conjunction with the Cell line Nucleofector™ Kit V reagent kit (Amaxa) according to the manufacturer's recommended protocols for the various cell lines. The cells were transfected with 100 nM siGENOME™ siRNA Human PIGN, (D-012463-01, Dharmacon) or 100 nM siGENOME™ Control siRNA Non-targeting siRNA #2, (D-001210-02-05, Dharmacon) and incubated for 24-72 hours. CRISPR/Cas9 experiments were conducted according to a modified LentiCRISPRv2 (Addgene plasmid #49535) protocol [8]. The gRNA (AAACGGTCATGTAGCTCTGATAGC) we employed targets *PIGN* at exon 4 and results in a frameshift [9]. The Lentiviral transduced CD34+ mononuclear cells were harvested for downstream applications 9 days post-infection.

### Western blot analyses

Cells were lysed in RIPA lysis buffer (Sigma-Aldrich) with protease and phosphatase inhibitor cocktails (Sigma). Protein concentration was determined by Bradford Assay (Life Technologies). Total protein lysate (15-40 µg) was separated in a NuPAGE™ 4-12% Bis-Tris Gel (Life Technologies) and transferred to Immun-Blot® PVDF Membrane (Bio-Rad). The blots were incubated overnight with anti-PIGN antibodies (HPA039922, Atlas Antibodies; and L-20: sc-85103, Santa Cruz); anti-MAD1 antibodies (Clone BB3-8, MABE867, Millipore), anti-Histone H2A.X (D17A3, Cell Signaling Technology) anti-phospho-Histone H2A.X ser139 (S139, Cell Signaling Technology) and HA-Tag (C29F4, Cell Signaling Technology) in TBS-T with 5% Non-Fat Dry Milk. The blots were subsequently probed with a horse radish peroxidase conjugated goat anti-rabbit IgG (AP132P, Millipore) or goat anti-mouse IgG antibody (AP124P, Millipore) and developed by ECL Prime (Amersham). Mouse beta-actin (C-4, sc-47778, Santa Cruz Biotechnology) was used as loading control.

### HA tag IP/Co-IP analyses

PIGN-HA IP/Co-IP experiments were conducted by transient transfection of CRISPR/Cas9 PIGN knockout HEK293 cells with the SRα promoter-driven expression vector pME Puro 3HA hPIGN or the empty vector without PIGN cDNA cloned [9]. The cells were transfected with 5 µg of the vector using the Lipofectamine 3000 transfection reagent (Life Technologies) according to the manufacturer's protocol. Protein samples were obtained 48 hours post transfection and 250 µg protein was used with the HA Tag IP/Co-IP Kit according to the manufacturers protocol. The eluates and 10% of the input lysates were used for Western blot analyses.

### TP53 sequence analyses

One microgram of DNA isolated from non-leukemic cells and leukemic cells was amplified using primers covering exon 2-11 of the TP53 gene including intron/exon boundaries according to instructions in the IARC database [10]. Each sample (non-leukemic and leukemic) was run in 7 PCR reactions. Each PCR product was spin column purified and sequenced using the 3130XL Genetic Analyzer (ABI systems) with the same primers in both reverse and forward directions. FinchTV version 1.4.0 and Nucleotide BLAST were used for sequence analyses [11]. Sequencing results were aligned to the TP53 GenBank sequence NC_000017.9 and sequence alterations were partly identified via manual inspection. Sequence alterations were aligned with the coding sequence of the TP53 protein and the impact on protein was determined using the IARC database (R18, April 2016) (http://p53.iarc.fr/p53Sequence.aspx) and Sorting Intolerant From Tolerant (SIFT) [10, 12].

### Cell cycle block/synchronization

Cell synchronization at the Go/G1, S and G2/M phases were conducted based on modified protocols [13].

Cell synchrony was monitored using propidium iodide-stained cells with the FACS Calibur flow cytometer (BD Biosciences). Protein lysates were obtained and used for Western blot analyses as earlier described. Please see supplementary figures for schematic of the cell block/synchronization procedures.

### Immunofluorescence and confocal microscopy

For missegregation and co-localization analyses, cells were blocked in early S-phase via the double thymidine treatment and released for 6-8 hours into the mitotic phase. Adherent cells were cultured on chambered slides. Cytospin was used to fix suspension cells on to the slides. Cells were fixed in 4% paraformaldehyde in PBS and permeabilized in -20°C 100% methanol. The slides were blocked in 2.5% normal goat serum diluted in PBS. For all wash steps, the cells were washed three times for 5 mins each in PBS. The cells were treated at 4°C overnight with primary antibodies: Human anti-centromere (kinetochore) (15-234, Antibodies Incorporated), PIGN (HPA039922, Atlas Antibodies) and MAD1 (Clone BB3-8, MABE867, Millipore) followed by washing and treatment for 2 hours at room temperature in the dark with secondary antibodies (1:1000): Alexa Fluor® 647 goat anti-human IgG (H+L) (A21445, Life Technologies), Goat anti-rabbit IgG, Dylight® 488 Conjugated Highly cross-adsorbed (35553, Life Technologies) and Goat anti-mouse IgG, Dylight™ 633 Conjugated (35513, Life Technologies) respectively. Antibodies were diluted in 1% normal goat serum diluted in PBS. The slides were partially dried in the dark and mounted in Vectashield Hard Set™ mounting medium with DAPI (H-1500, Vector Laboratories). Images were acquired using the Leica SP8 Inverted confocal microscope at the Microscopy Imaging Facility at Penn State College of Medicine. Three-dimensional image stacks were acquired in 0.15-μm steps using a ×40 1.4 N.A oil immersion objective. Deconvolution and analyses of image stacks were performed using the Huygens workstation (Scientific Volume Imaging B.V.) and the Imaris Microscopy Image Analysis software (Bitplane AG).

### Bioinformatics analyses and statistical analyses

The GENE-E (http://www.broadinstitute.org/cancer/software/GENE-E/) matrix visualization and analysis platform was used to generate a heat map of the CIN-70 gene expression profiles of the CD34+ cells of 55 MDS patients and 11 healthy controls utilizing data generated on the Affymetrix GeneChip U133 Plus2.0 platform from the study GSE4619 [14].

The randomForest v4.6-12 R package with default parameters was used in a Random Forest Analysis to classify patients based on the following MDS risk stratification: control, RA, RARS, RAEB1 and RAEB2. The importance of each gene (predictor) was calculated using the package's mean decrease in accuracy, which measures how much the model fit decreases when a variable is dropped.

In RNA-seq Analysis study, raw RNA-seq files for the dbGAP study phs001027.v1.p1 were downloaded as sequence raw archive (SRA) files and then converted to FASTQ, using the SRA toolkit version 2.5.4. The RNA-seq reads were aligned to the most recent reference genomes (hg38) using Tophat (v2.0.9) by allowing up to 2 mismatches [15]. The junction files data analyses were loaded on to IGV (Integrated Genome Viewer) to survey intronic frame shift in *PIGN* genes.

GraphPad Prism 5 software and Microsoft Excel 2010 were used for statistical analyses. Two-tailed Student's t-tests and One-way ANOVA followed by Tukey's post hoc tests were used for comparisons. p-values $\leq 0.05$ were considered statistically significant.

## REFERENCES

1. Arber DA, Orazi A, Hasserjian R, Thiele J, Borowitz MJ, Le Beau MM, Bloomfield CD, Cazzola M, Vardiman JW. The 2016 revision to the World Health Organization classification of myeloid neoplasms and acute leukemia. Blood. 2016; 127: 2391-405. doi: 10.1182/blood-2016-03-643544.
2. Swerdlow SH, Campo, E, Harris, NL, Jaffe, ES, Pileri, S.A, Stein, H, Thiele, J, Vardiman, JW. WHO classification of tumours of haematopoietic and lymphoid tissues. Fourth Edition. 2008.
3. Pu JJ, Hu R, Mukhina GL, Carraway HE, McDevitt MA, Brodsky RA. The small population of PIG-A mutant cells in myelodysplastic syndromes do not arise from multipotent hematopoietic stem cells. Haematologica. 2012; 97: 1225-33. doi: 10.3324/haematol.2011.048215.
4. Sandes AF, Kerbauy DM, Matarraz S, Chauffaille Mde L, Lopez A, Orfao A, Yamamoto M. Combined flow cytometric assessment of CD45, HLA-DR, CD34, and CD117 expression is a useful approach for reliable quantification of blast cells in myelodysplastic syndromes. Cytometry B Clin Cytom. 2013; 84: 157-66. doi: 10.1002/cyto.b.21087.
5. Hasanali ZS, Saroya BS, Stuart A, Shimko S, Evans J, Vinod Shah M, Sharma K, Leshchenko VV, Parekh S, Loughran TP, Jr., Epner EM. Epigenetic therapy overcomes treatment resistance in T cell prolymphocytic leukemia. Sci Transl Med. 2015; 7: 293ra102. doi: 10.1126/scitranslmed.aaa5079.
6. Stothard P. The sequence manipulation suite: JavaScript programs for analyzing and formatting protein and DNA sequences. Biotechniques. 2000; 28: 1102, 4.

7. Adzhubei IA, Schmidt S, Peshkin L, Ramensky VE, Gerasimova A, Bork P, Kondrashov AS, Sunyaev SR. A method and server for predicting damaging missense mutations. Nat Methods. 2010; 7: 248-9. doi: 10.1038/nmeth0410-248.
8. Sanjana NE, Shalem O, Zhang F. Improved vectors and genome-wide libraries for CRISPR screening. Nat Methods. 2014; 11: 783-4. doi: 10.1038/nmeth.3047.
9. Ohba C, Okamoto N, Murakami Y, Suzuki Y, Tsurusaki Y, Nakashima M, Miyake N, Tanaka F, Kinoshita T, Matsumoto N, Saitsu H. PIGN mutations cause congenital anomalies, developmental delay, hypotonia, epilepsy, and progressive cerebellar atrophy. Neurogenetics. 2014; 15: 85-92. doi: 10.1007/s10048-013-0384-7.
10. Petitjean A, Mathe E, Kato S, Ishioka C, Tavtigian SV, Hainaut P, Olivier M. Impact of mutant p53 functional properties on TP53 mutation patterns and tumor phenotype: lessons from recent developments in the IARC TP53 database. Hum Mutat. 2007; 28: 622-9. doi: 10.1002/humu.20495.
11. Altschul SF, Gish W, Miller W, Myers EW, Lipman DJ. Basic local alignment search tool. J Mol Biol. 1990; 215: 403-10. doi: 10.1016/S0022-2836(05)80360-2.
12. Ng PC, Henikoff S. Predicting deleterious amino acid substitutions. Genome Res. 2001; 11: 863-74. doi: 10.1101/gr.176601.
13. Jackman J, O'Connor PM. Methods for synchronizing cells at specific stages of the cell cycle. Curr Protoc Cell Biol. 2001; Chapter 8: Unit 8 3. doi: 10.1002/0471143030.cb0803s00.
14. Pellagatti A, Cazzola M, Giagounidis AA, Malcovati L, Porta MG, Killick S, Campbell LJ, Wang L, Langford CF, Fidler C, Oscier D, Aul C, Wainscoat JS, et al. Gene expression profiles of CD34+ cells in myelodysplastic syndromes: involvement of interferon-stimulated genes and correlation to FAB subtype and karyotype. Blood. 2006; 108: 337-45. doi: 10.1182/blood-2005-12-4769.
15. Trapnell C, Pachter L, Salzberg SL. TopHat: discovering splice junctions with RNA-Seq. Bioinformatics. 2009; 25: 1105-11. doi: 10.1093/bioinformatics/btp120.

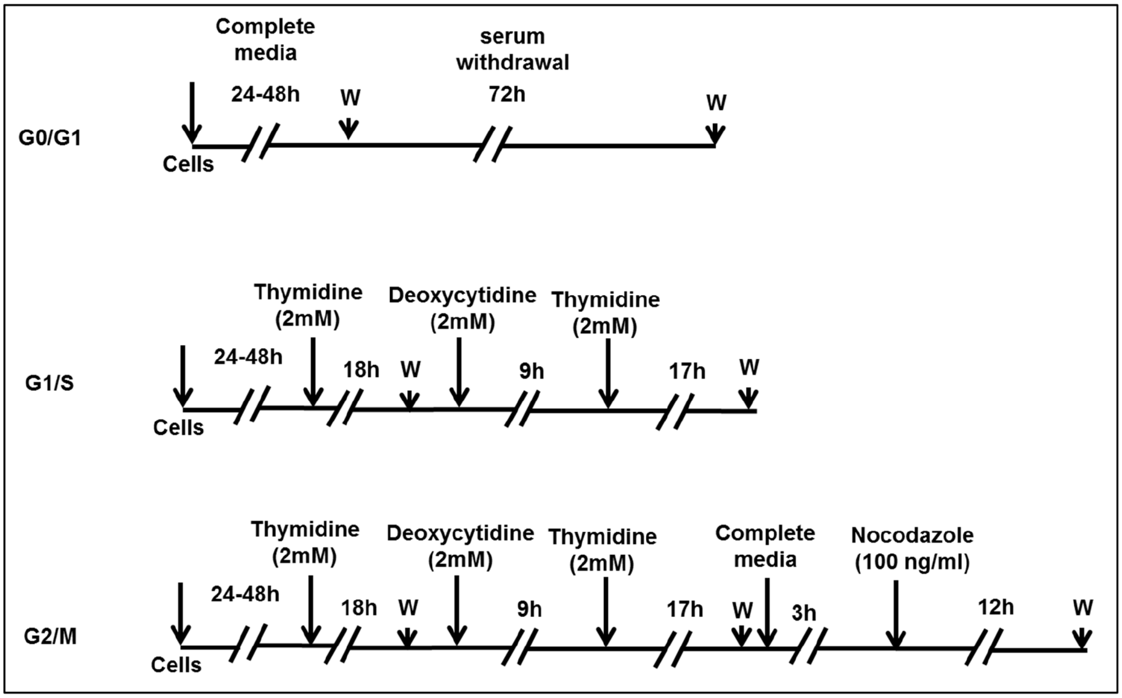


**Supplementary Figure 1: Schematic procedure employed for cell cycle synchronization experiments in HL60 and K562 cell lines.** W= wash step.

**Supplementary Table 1: Samples with intron fragment retention between exons 14 and 15**

| # | Sample Description | Dx Status |
|---|---|---|
| **M1** | Leukemic sorted M1 cells | AML-MRC |
| **M2** | Leukemic sorted M2 cells | AML-MRC |
| **MDS-L** | MDS-L cell line | MDS transformed to AML |
| **M4** | Mononuclear cells rich in blast from M4 | AML-MRC |
| **1** | AML003 Dx | AML[a] |
| **2** | AML139Dx | AML[a] |
| **3** | AML028 Dx | AML[a] |
| **4** | AML059 Dx | AML[a] |
| **5** | AML125 Dx | AML[a] |
| **6** | AML 117 Dx | AML[a] |
| **7** | AML103 Rel | relapsed AML[a] |
| **8** | AML128 Dx | AML [a] |
| **9** | AML 051 Dx | AML[a] |
| **10** | AML107 Rel | relapsed AML[a] |
| **11** | AML117 Rel | relapsed AML [a] |

[a]. RNA-seq junction file data analyses from dbGAP phs001027.v1.p1.

**Supplementary Table 2: Primers covering the coding sequence of PIGN**

| Gene | Exon[a] | Forward Primer 5'-3' | Reverse Primer 5'-3' | (bp) | Product Position |
|---|---|---|---|---|---|
| *PIGN* | 3-8 | TTCTTCGCCTCCATCTTTGACA | GAACCCCAGTCTGTCATTCCAT | 767 | 414-1180 |
| | 8-16 | ATGGAATGACAGACTGGGGTTC | GACAGGCTTGAATCAGCAGAAA | 726 | 1159-1884 |
| | 16-19 | AAGCCATCTCCTGCCTTGTA | AGATGTCTGGCTTTCGACCT | 413 | 1811-2223 |
| | 19-29 | TGTAGGTCGAAAGCCAGACATC | AGCTGCCATAATCCTTGACCAA | 824 | 2201-3024 |

[a] Exon and product positions are based on PIGN mRNA transcript variant 2 NM_012327.5.

**Supplementary Table 3: qRT-PCR primer sequences used to compare gene expression in AML patient during leukemia active phase and remission phase**

| Gene | Forward primer 5'-3' | Reverse primer 5'-3' |
|---|---|---|
| *SIRT1* | GGGGCTGCGGTTCCTACTG | GTCCAGTCACTAGAGCTTGCAT |
| *H2AX* | GTCGTGCTTCACCGGTCTAC | TCAGCGGTGAGGTACTCCAG |
| *DR5* | CTCCTTTTCTGCTTGCGCTG | TGATGCCTGATTCTTTGTGGAC |
| *BAXα* | CGGGGACGAACTGGACAGTAAC | GCGTCCCAAAGTAGGAGAGGA |
| *SAE2* | AGCTGCCCGAAACCATGTTA | TCTGGGTCGGCTTAGGATGA |
| *p21* | AGAAGAAGTCTGCTGGTCACAGCG | CTGGACCTTTCCGGGCCGTG |
